# A novel and easily reproducible method for reestablishing pneumoperitoneum during laparoscopic colorectal resectional surgery

**DOI:** 10.1308/003588412X13171221591259f

**Published:** 2012-05

**Authors:** M Mobasheri, K Khatri, M Gudgeon, P Sains

**Affiliations:** ^1^Kingston Hospital NHS TrustUK; ^2^Western Sussex Hospitals NHS TrustUK; ^3^Frimley Park Hospital NHS Foundation TrustUK

## BACKGROUND

Laparoscopic assisted surgery for the resection of colorectal cancer is fast becoming the norm.^1^ Following mobilisation for left-sided resections, many surgeons deliver the colon via an extended 5cm umbilical port site incision. Once the extracorporeal component of the surgery has been completed, pneumoperitoneum must be re-established and a working umbilical port reinserted to fashion the stapled anastomosis and check haemostasis. We describe a novel and easily reproducible method of achieving this.

## TECHNIQUE

**Figure 1:**
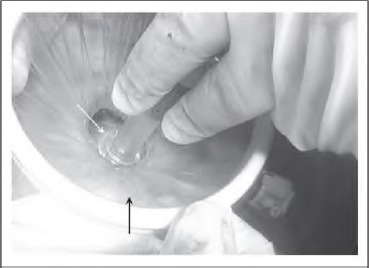
The port has been introduced through the unrolled wound protector (black arrow) and the balloon inflated (white arrow).

**Figure 2:**
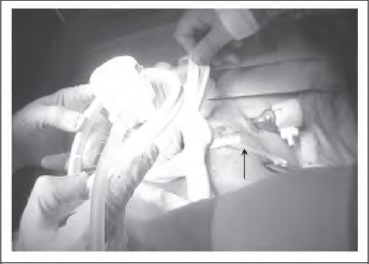
The medium swab is secured around the wound protector and port. Note that the nylon tape has already been tied preceding this (ends of the tape shown by the black arrow).

**Figure 3:**
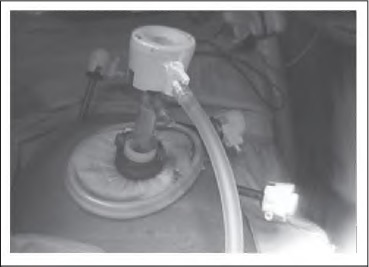
The wound protector has been re-secured, ensuring the medium swab is tucked underneath so that an airtight seal is achieved. Carbon dioxide insufflation and pneumoperitoneum have been re-established.

Once the colon has been returned into the peritoneal cavity, the Alexis® (Applied Medical Resources Corporation, Rancho Santa Margarita, CA, US) wound retractor (used to protect the wound during the extracorporeal component of the surgery) is unrolled but not removed. The 10mm balloon tipped port (used for the initial laparoscopic component of the surgery) is reinserted and the balloon inflated (Fig 1). Next, a length of nylon tape is tied around the wound protector and port to prevent the latter slipping out. A medium swab is tied over the nylon tape (Fig 2). Finally, the wound protector is re-secured against the abdominal wall and the pneumoperitoneum re-established (Fig 3).

## DISCUSSION

Many techniques for re-establishing pneumoperitoneum, including the use of sealing devices,^2^·^3^ do not allow reinsertion of a working port. Interrupted closure with insertion of a port between sutures results in a poorly secured port that can easily slip out, much to the surgeon’s frustration. Furthermore, open re-entry (which is occasionally required) necessitates cutting of these sutures and wastes time. Our technique requires no specialist equipment and allows rapid open reentry into the abdomen should this be needed. We have encountered no problems with this method.
